# Endurance training-induced increase in muscle oxidative capacity without loss of muscle mass in younger and older resistance-trained men

**DOI:** 10.1007/s00421-021-04768-4

**Published:** 2021-08-14

**Authors:** Paul William Hendrickse, Tomas Venckunas, Justinas Platkevicius, Ramutis Kairaitis, Sigitas Kamandulis, Audrius Snieckus, Arvydas Stasiulis, Jolanta Vitkiene, Andrejus Subocius, Hans Degens

**Affiliations:** 1 Research Centre for Musculoskeletal Science and Sports Medicine, Department of Life Science, Manchester Metropolitan University, John Dalton Building, Chester Street, Manchester, M1 5GD UK; 2 Lithuanian Sports University, Kaunas, Lithuania; 3 Clinic of Radiology, Republican Hospital of Kaunas, Kaunas, Lithuania; 4 Department of Surgery, Kaunas Clinical Hospital, Kaunas, Lithuania; 5 Clinic of Surgery, Republican Hospital of Kaunas, Kaunas, Lithuania

**Keywords:** Capillarisation, Muscle size

## Abstract

While concurrent training is regularly used in older populations, the inverse relationship between fibre size and oxidative capacity suggests that endurance training in resistance-trained individuals may result in some loss of resistance training-induced gains in muscle mass, which may be more pronounced in older people. We investigated the impact of superimposed endurance training in younger (28.5 ± 4.8 years; *n* = 8) and older (67.5 ± 5.5 years; *n* = 7) highly resistance-trained men. Participants underwent a 10-week endurance cycling training programme consisting of five 6-min intervals at 75% max heart rate (HRmax) separated by 4-min intervals at 90% HRmax. The anatomical cross-sectional area (ACSA) of the thigh muscles, as determined with MRI, was 24% smaller in older compared to younger participants (*p* < 0.001). Although maximal oxygen consumption (VO_2_max) was also lower in the older group (*p* < 0.001), VO_2_max per kg body mass did not differ significantly between younger and older participants. Histological analyses of biopsies of the *m. vastus lateralis* showed that endurance training induced an increase in succinate dehydrogenase activity in both younger and older participants (*p* ≤ 0.043), and an increase in the number of capillaries around type I fibres (*p* = 0.017). The superimposed endurance training did not induce a significant decrease in thigh ACSA, fibre cross-sectional area, or knee extensor maximum voluntary isometric force. These observations indicate that adding endurance training to resistance training can lead to positive endurance-related adaptations without negative consequences for muscle size and strength in older and younger resistance-trained people.

## Introduction

The combination of resistance and endurance training, known as concurrent training, is regularly prescribed to older people to promote increases in muscle mass, strength and endurance (Cadore et al. 2014). Resistance exercise promotes muscle hypertrophy and a concomitant increase in strength, while endurance exercise induces, among other adaptations, increases in stroke volume, muscle oxidative capacity and angiogenesis with a reduction in type II fibre cross-sectional area (FCSA), resulting in an increase in whole body maximal oxygen uptake (VO_2_max) (Baar 2006). While improvements in both strength and endurance are beneficial in older people, it is thought that adding endurance training to resistance exercise blunts the adaptation to resistance exercise and vice versa. In 1980, Hickson coined the term “interference effect”, now known as the concurrent training effect (CTE), to describe the blunted adaptation to resistance training in concurrent trained subjects when compared to those who only performed resistance training. At the level of the muscle, this is illustrated by the observation that inclusion of high-intensity endurance training attenuated the resistance training-induced hypertrophy of type I fibres, and that high-intensity endurance exercise alone led to reductions in type I fibre size (Kraemer et al. 1995). This incompatibility of training modalities may, however, be due to a high frequency and/or intensity of the superimposed training modality. In line with this notion, Hickson’s study was notably very high in volume, frequency and intensity, likely resulting in non-functional overreaching (Hickson 1980), and (McCarthy et al. 1995) demonstrated that combining resistance and endurance training in untrained males induced comparable adaptations to each modality alone when the frequency and intensity of training are moderate.

It is thought that the inverse relationship between muscle fibre size and oxidative capacity is a consequence of oxygen, ADP and ATP diffusion limitations that constraint fibre size (van Wessel et al. 2010; Degens 2012; Kinsey et al. 2007; van der Laarse et al. 1998). There thus seems to be a trade-off (van der Laarse et al. 1998) between endurance (and high oxidative capacity) and power or force-generating capacity (large fibre size) of a muscle fibre that may explain the CTE (van Wessel et al. 2010).

The effect of superimposed endurance training in both younger and older resistance-trained individuals is yet to be determined. It has been shown, however, that endurance training combined with resistance exercise may not diminish the strength gain of resistance training (Petre et al. 2018) and animal models have shown that the fibre size constraint can be broken. For instance, oestrogen-related receptor gamma (Errγ) overexpression in myostatin null mice exhibits as large muscle fibres as the myostatin null mice despite having a higher oxidative capacity (Omairi et al. 2016), and functional overload resulted in both hypertrophy and an increased oxidative capacity even in older mice (Ballak et al. 2016). In both cases, this was associated with a denser capillary bed, suggesting that angiogenesis is instrumental in breaking the size constraint. However, no systematic study has yet investigated whether also in humans with larger muscle fibres than rodents (Wust et al. 2009) the trade-off between fibre size and oxidative capacity can be overcome, and in particular whether superimposing endurance exercise will negate the gains in muscle fibre size in long-term resistance-trained people.

Such challenges to adaptation to a superimposed endurance training regime in long-term resistance-trained people may be particularly problematic in older people who already suffer from age-related decreases in muscle mass, strength, capillarisation, oxidative capacity and fatigue resistance, and impaired adaptations to hypertrophic and endurance stimuli in both humans and rodents (Ballak et al. 2016; Conley et al. 2000; Degens and Alway 2003; Petrella et al. 2006; Walters et al. 1991; Hendrickse et al. 2020). In addition, while concurrent training improved strength similarly to resistance training in ageing men, the addition of endurance training prevented hypertrophy of type II fibres (Karavirta et al. 2011). However, other studies have found that combined training in untrained older people increases muscle size similarly to resistance training only (Holviala et al. 2012; Sillanpää et al. 2008).

The potential detrimental effect of endurance training on the muscle (fibre) size of highly resistance-trained men may be even more pronounced in older highly resistance-trained men, if, like in overloaded mouse muscle, the angiogenic response is attenuated in older adults (Ballak et al. 2016; Degens and Alway 2003; Hendrickse et al. 2020).

The aim of the present study was to assess the impact of superimposing endurance training onto the usual resistance training programmes of both younger and older highly resistance-trained men on (1) the oxidative capacity and (2) size of the muscle fibres, and (3) the number of capillaries around a fibre (CAF). Given that CTE suggests that the hypertrophic response is attenuated in concurrent compared to resistance training alone, and based on the size principle of striated muscle cells (van der Laarse et al. 1998) we hypothesised that superimposing endurance training in both older and younger highly resistance-trained men will lead to an increase in fibre oxidative capacity and a decrease in fibre size. This decrease in fibre size will be more pronounced in older highly resistance-trained men due to an attenuated angiogenic response. If endurance exercise does induce an increase in muscle oxidative capacity without compromising fibre size, it shows both training programmes can be used concurrently to enhance both muscle strength and endurance capacity.

## Methods

The Kaunas Regional Biomedical Research Ethics Committee (Authorisation number BE-10-4) provided ethical approval for the study. All subjects provided informed consent prior to participation.

### Subjects

Eight highly resistance-trained younger (28.5 ± 4.8 years) and seven older (67.5 ± 5.5 years) men engaged in regular resistance training were recruited. Participants were either bodybuilders in their offseason, retired bodybuilders or men that resistance-trained for recreational purposes. All participants performed at least 2 upper body and 2 lower body resistance training sessions per week. Younger participants had performed regular resistance training for a minimum of 5 years and older participants for a minimum of 20 years. The highly trained status of our resistance-trained subjects is reflected by their higher than age-expected maximal voluntary quadriceps torque (354 Nm *vs.* 220 Nm for younger and 225 Nm *vs*.149 Nm for older men, respectively) (Lindstrom et al. 1997). In addition, the fat free mass index (FFMI) (see Table 1) was within the top 5% and 10% in younger and older men, respectively, further indication of skeletal muscle hypertrophy according to Schutz et al. (2002).

**Table 1:** Participant characteristics, maximal voluntary isometric knee extension contraction torque (MVC) and maximal oxygen uptake (VO_2_max)

Variables	Young (*n* = 8)		Old (*n* = 7)	
	Pre	Post	Pre	Post
Age (y)	28.5 ± 4.8 (23–35)		67.5 ± 5.5 (61–77)	
Training experience (y)	10.6 ± 5.1 (5–20)		36.7 ± 12.1 (20–50)	
Height (cm)	182 ± 5 (176–188)		175^a^ ± 7 (162–180)	
Body mass (kg)	98.9 ± 7.6 (89.4–109.4)	101.0 ± 8.7 (85.9–110.7)	83.0^a^ ± 8.1 (68.5–93.2)	82.8^a^ ± 8.0 (69.0–93.0)
BMI (kg·m^−2^)	30.4 ± 2.1 (27.3–34.5)	30.5 ± 2.4 (26.8–34.1)	27.2^a^ ± 1.1 (26.1–28.8)	27.2^a^ ± 0.9 (26.0–28.7)
Fat mass (%)	18.0 ± 6.3 (9.7–31.7)	18.2 ± 6.0 (8.5–29)	22.3 ± 3.4 (19.0–27.9)	22.1 ± 3.3 (19.0–28.0)
FFMI (kg·m^−2^)	24.7 ± 0.84 (23.5–26.1)	25.1 ± 1.00 (23.6–26.8)	21.1 ± 0.31 (20.7– 21.6)	21.0 ± 0.374 (20.5–21.7)
Thigh subcutaneous fat (%)	20.4 ± 5.0 (12.7–31.5)	19.8 ± 4.6 (8.3–24.0)	22.4 ± 3.6 (17.6–30.0)	21.7 ± 3.0 (18.3–27.0)
Thigh muscle ACSA (cm^2^)	242 ± 18 (207–262)	244 ± 13 (220–267)	183^a^ ± 16 (161–210)	190^a^ ± 17 (163–211)
VO_2_max (L·min^−1^)	3.24 ± 0.48 (2.56–3.60)	3.41 ± 0.27 (2.99–3.79)	2.68^a^ ± 0.39 (2.03–3.15)	2.59^a^ ± 0.27 (2.28–3.00)
VO_2_max (mL min^−1^ kg^−1^)	32.2 ± 5.3 (25.4–39.5)	33.9 ± 3.0 (29.8–38.1)	33.1 ± 7.2 (24.4–42.0)	31.8 ± 4.7 (28.1–39.5)
HRmax (bpm)	179 ± 11 (163–194)	176 ± 8 (168–191)	151^a^ ± 12 (136–165)	150^a^ ± 7 (140–157)
Power_max_ (W)	356 ± 48 (270–430)	377 ± 20 (360–415)	292 ^a^ ± 42 (235–345)	301 ^a^ ± 32 (260–330)
Power_max_/BM (W kg^−1^)	3.54 ± 0.57 (2.74–4.39)	3.76 ± 0.41 (3.34–4.42)	3.58 ± 0.66 (2.67–4.47)	3.70 ± 0.50 (3.07–4.22)
Fibre form factor	1.32 ± 0.04 (1.24–1.37)	1.33 ± 0.04 (1.27–1.40)	1.27 ± 0.08 (1.13–1.38)	1.28 ± 0.08 (1.13–1.44)

Data are mean ± standard deviation, and range in parenthesis

*BMI* body mass index, *FFMI* fat free mass index, *ACSA* anatomical cross-sectional area, *HRmax* maximal heart rate, *VO*_*2*_*max* maximal oxygen consumption, *Power*_*max*_ power at VO_2_max, *Power*_*max*_/BM Power_max_ per body mass

^a^Indicates a significant difference from young participants (*p* < 0.05)

### Experimental design

The volunteers participated in a 10-week endurance-training programme that was superimposed on their usual training programme. Although participants did not follow a prescribed resistance-training regimen, all performed resistance exercise at least 4 times per week (2 sessions for upper body, 2 sessions for lower body). Participants had not performed regular endurance exercise before the study began. Before and after the training programme, body fat percentage was determined, an MRI scan of the upper leg was performed and a *vastus lateralis* muscle biopsy was taken. In addition, the maximal oxygen consumption (VO_2_max) and the maximal voluntary isometric contraction torque (MVC) of the knee extensor muscles were measured.

### Endurance training programme

The endurance-training programme was home-based but preceded by a supervised familiarisation session in the laboratory. The participants had to provide written comments on each training session. These reports indicated at least a 90% completion of the set number of endurance training sessions. Exercise intensity was monitored with a heart rate monitor.

The participants were instructed to perform cycle ergometry 3 times per week with a protocol similar that used by (McPhee et al 2011) that has been shown to increase muscle oxidative capacity. To ensure adherence, they were encouraged to carry out the additional endurance training at a convenient time, either in combination with their resistance training during the same gym visit, as a separate session during the same day, or at different day from resistance training. When both modalities were performed on the same day or within the same session, resistance training was always completed first. The intensity of the endurance-training was increased during the first 6 weeks from 45 min cycling at 75% of the maximal heart rate (HRmax) in the first week to 5 intervals of 6 min cycling at 75% HRmax interspersed with 4 min cycling at 90% HRmax in the final 4 weeks.

### Anthropometric and MRI analyses

**Fig. 1:**
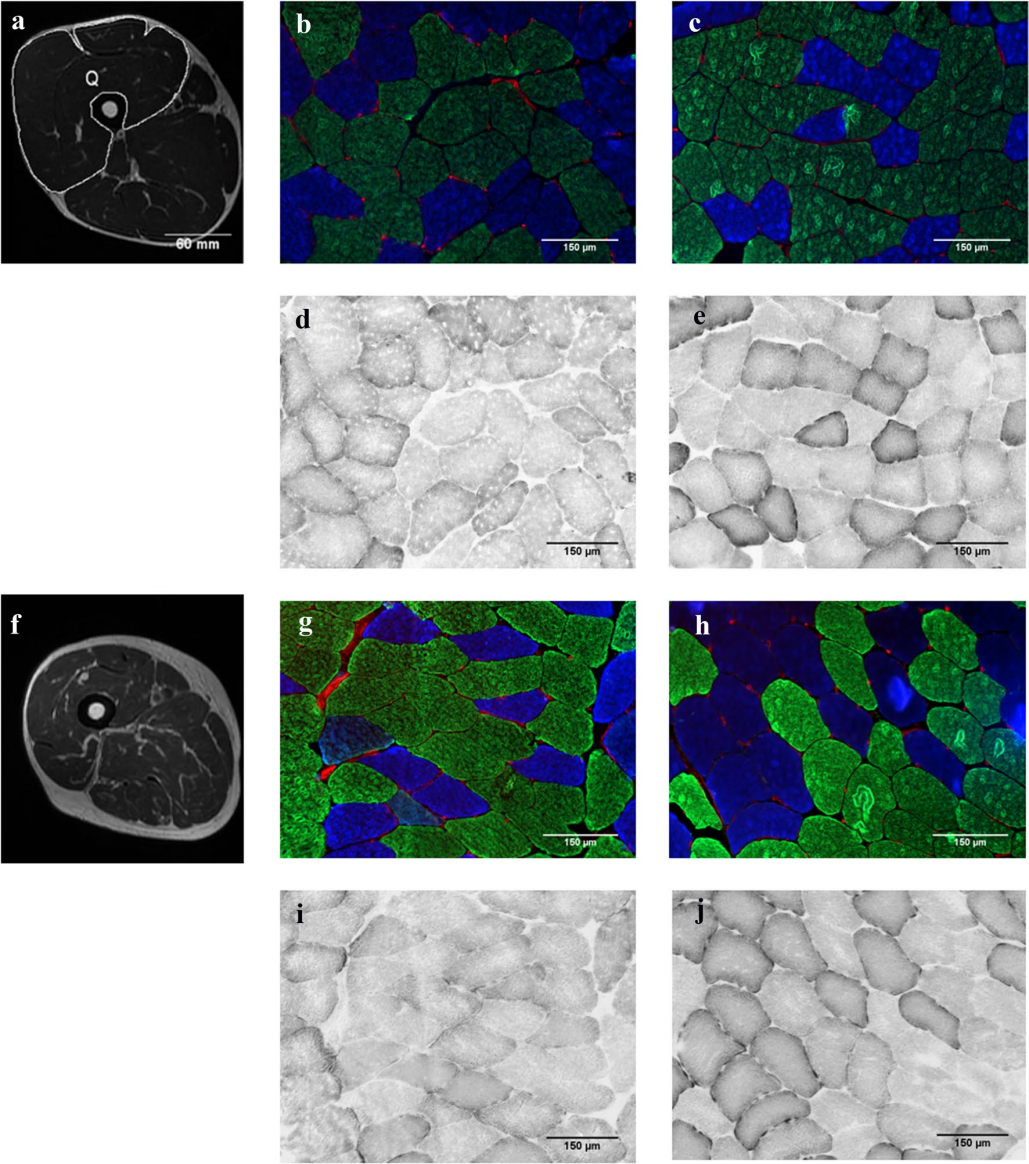
**a** and **f** show MRI scans from younger and older participants, respectively, of a left thigh at approximately 60% of femur length (from distal end) with labelled quadriceps muscles in the image of the younger participant (Q). **b**, **c**,** g** and **h** show muscle cross-sections from younger pre, younger post, older pre and older post, respectively, immuno-fluorescently stained for type I (blue fibres) and type II (green and non-stained fibres) fibres, and capillaries (stained red) stained with Rhodamine-labelled *Ulex Europaeus* Agglutinin I. **d**, **e**, **i** and **j** show serial sections of (**b**), (**c**), (**g**) and (**h**), respectively, all stained for succinate dehydrogenase activity

Body fat percentage was measured using bioelectrical impedance (Tanita, Tokyo, Japan). Images of the thigh were taken using a 1.5 T MRI scanner (Signa Explorer, GE Health Care, China). With the participant in a supine position, a Cor FSE protocol was used and multiple 4-mm thick serial transverse sections were taken along the length of the thigh with no inter-slice gap. Images were analysed using ImageJ (Rasband, W.S., ImageJ, U. S. National Institutes of Health, Bethesda, Maryland, USA, http://imagej.net/Downloads). At 60% femur length (from distal), the total cross-sectional area of the muscle tissue was measured. The optical density (OD) of the muscle was measured as an indication of fat content. Figure 1a shows an example of an MRI image of the thigh.

### *Maximal voluntary contraction (MVC) and maximal oxygen consumption (VO*_*2*_*max)*

On a separate day, MVC and maximal oxygen uptake (VO_2_max) were measured. After a 10-min warm up on the cycle ergometer, the MVC was measured using an isokinetic dynamometer (System 3; Biodex Medical Systems, Shiley, New York) at a knee angle of 50°, 70° and 90° (0° = anatomical zero/full leg extension) for 2 s with 60 s rest in between in a random sequence. Subjects were seated upright in the dynamometer chair with double shoulder seat belts stabilizing the upper body and were encouraged to perform each contraction as hard as possible. Pre- and post-intervention testing was performed at the same time of day and participants were asked to abstain from food for 2 h prior to testing.

To assess VO_2_max, participants cycled at 40 W for 3 min, after which the load was increased by 5 W every 10 s while maintaining a pedalling rate of around 70 rpm. A portable breath-by-breath analyser (Oxygen Mobile; Jaeger/VIASYS Healthcare, Hoechberg, Germany) was used and heart rate was monitored during the test (S-625X; Polar Electro, Kempele, Finland). Participants were required to continue cycling until their heart rate was at least 90% of the predicted HRmax and the respiratory exchange ratio > 1.1. VO_2_max and HRmax were determined as the highest 15-s averaged values during the last minute of the test.

### Histological analyses

Biopsies of the *m. vastus lateralis* were taken under aseptic conditions and local anaesthesia with 2% lidocaine employing a conchotome after MRI imaging. The biopsies were frozen in isopentane cooled with liquid nitrogen and stored at − 80 °C until use.

Cross sections (10 μm) were cut at − 20 °C using a cryostat (Leica CM3050 S, Leica Microsystems, Nussloch, Germany). Sections were incubated in blocking solution (10% goat serum in phosphate-buffered saline (PBS)) for 60 min, then incubated for 120 min with BAD5 (3:100) and SC-71 (1:50) for type I and type IIa myosin heavy chain, respectively (Developmental Studies Hybridoma Bank, USA). After washing in PBS three times for 5 min, sections were incubated for 60 min in secondary antibodies Alexa Fluor 350 IgG2b for type I (1:500), Alexa Fluor 488 IgG1 for type IIa (Thermofisher Scientific, USA) and Rhodamine-labelled lectin *Ulex Europaeus* Agglutinin I (1:200) (Vector Laboratories, California, USA) to detect capillaries. After three 5-min washes in PBS, the slides were mounted using ProLong Diamond Antifade mountant (Thermofisher) and imaged at 10× magnification (Fig. 1b). Serial sections were stained for succinate dehydrogenase (SDH) as described previously (Hendrickse et al. 2020). The OD of the stain at 660 nm gives a quantitative indication of oxidative capacity (Fig. 1c) (van der Laarse et al. 1989). For each section, a calibration curve was created using a series of filters with known ODs to adjust for variation in background staining and lighting between sections. The OD of the SDH stain and fibre cross-sectional area (FCSA) of each fibre were determined using ImageJ. Also, the number of capillaries around a fibre (CAF) was determined for each fibre type. The form factor of the fibre was calculated as perimeter^2^/(4π·FCSA). A higher value of the form factor indicates a greater deviation from circularity (Barnouin et al. 2017). All image analyses were completed by the same investigator and a minimum of 50 fibres were analysed per biopsy.

### Statistics

All statistical analyses were completed with SPSS software. The Shapiro–Wilk test showed that all data were normally distributed. Repeated-measures analysis of variance (ANOVA) was used with pre- and post-exercise, fibre type (I vs. II) and knee angle (50°, 70° and 90°) as within factors, and age as a between factor. Three-way interactions were excluded. If interactions were found, Bonferroni-corrected post-hoc tests were done to locate differences. Differences were considered significant at *p* < 0.05.

## Results

### Participant characteristics

Table 1 shows that younger participants were taller (*p* = 0.042), had a higher body mass (*p* = 0.001) and BMI (*p* = 0.002) compared to older participants. There were no significant differences between younger and older men in body fat and subcutaneous fat percentage in the thigh area. While younger individuals had higher VO_2_max (L·min^−1^), HRmax (*p* < 0.001) and max power (W) (*p* ≤ 0.023) than older individuals, there was no significant difference between younger and older men in VO_2_max (mL·min^−1^·kg^−1^) and max power (W·kg^−1^) per kg body mass. Body mass, BMI, VO_2_max and power were not significantly changed with endurance training.

### Muscle properties

**Fig. 2:**
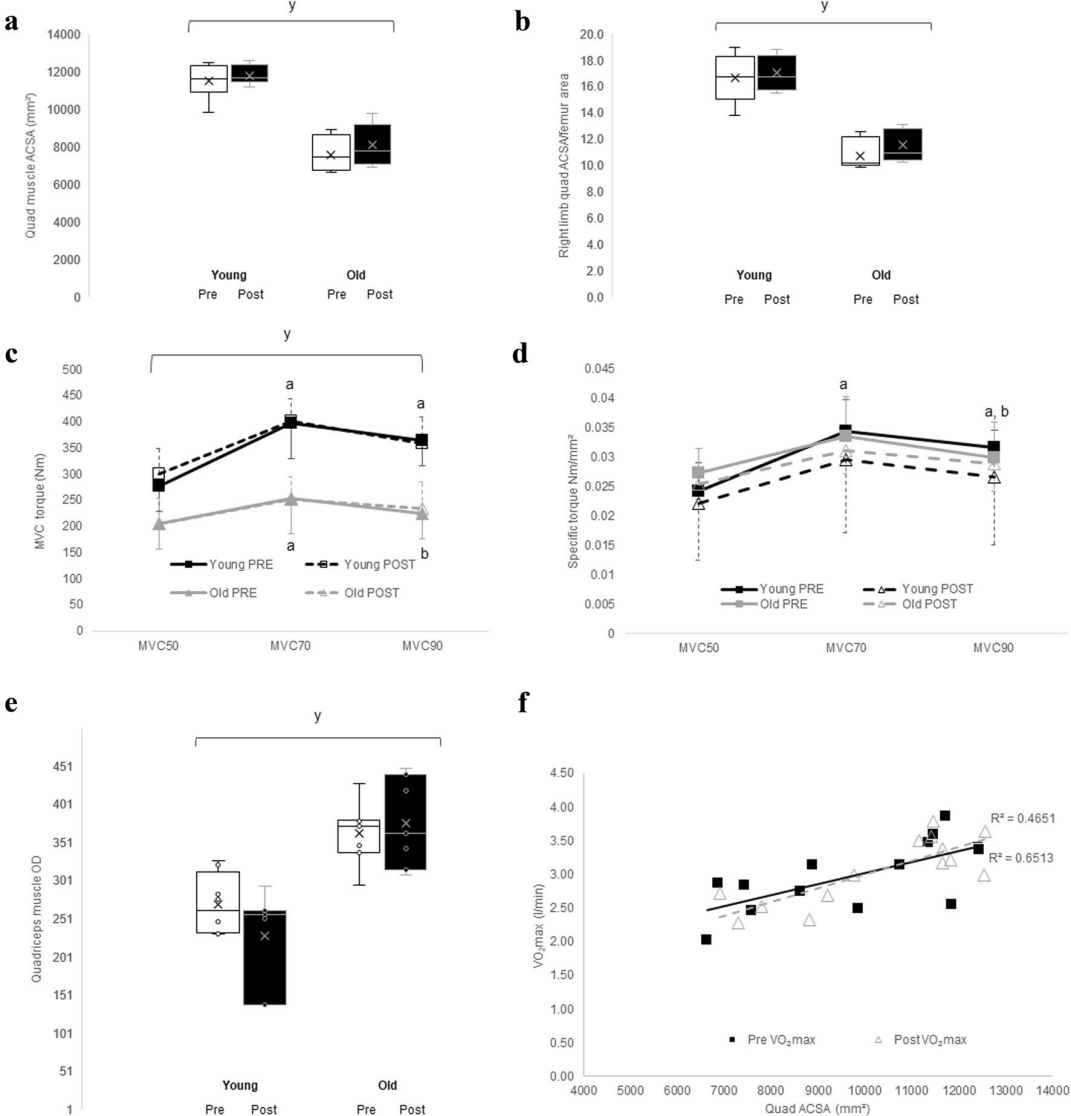
**a** Total quadriceps muscle anatomical cross-sectional area (ACSA) pre- and post-endurance training in young and old highly resistance-trained men. **b** Shows right quadriceps ACSA/femur area pre- and post-endurance exercise in young and old participants. **c** Shows maximal voluntary isometric contraction of knee extension (MVC) at 50, 70 and 90° (MVC50, MVC70 and MVC90, respectively) of young and old participants pre- and post-endurance exercise. **d** Shows the specific torque (MVC/quadriceps ACSA) of quadriceps muscles at MVC50, MVC70 and MVC90 for young and old participants pre- and post-endurance exercise. ^y^Indicates a significant difference to young participants at *p* < 0.001; ^a^indicates a significant difference to MVC50 at *p* ≤ 0.007. ^b^Indicates a significant difference to MVC70 at *p* ≤ 0.002. **e** Shows the quadriceps muscle optical density (OD) in young and old highly resistance-trained men pre- and post-endurance exercise programme. **f** Shows the relationship between quadriceps anatomical cross-sectional area (ACSA) and maximal oxygen consumption (VO_2_max) pre- and post-endurance exercise. ^y^Indicates a significant difference in OD compared to young subjects at *p* ≤ 0.03

The anatomical cross-sectional area (ACSA) of all muscles in the thigh (Table 1; *p* < 0.001) and quadriceps muscles (Fig. 2a; *p* < 0.001) were larger in the younger than older participants. The quadriceps ACSA/femur ratio was lower (*p* < 0.001) in older participants compared to younger (Fig. 2b). In addition, the MVC of younger participants was higher than that of the older at all angles (*p* < 0.001) (Fig. 2c). There was a significant angle × age interaction for MVC (*p* = 0.009). In younger participants, the MVC was greater at 70° and 90° than at 50° (*p* < 0.001 and *p* = 0.004, respectively). In older participants, the MVC at 70° was greater than at 50° (*p* = 0.005) and at 90° (*p* = 0.001). The specific torque of the extensor muscles (MVC/quadriceps ACSA, Fig. 2d) did not differ significantly between younger and older participants at any knee angle. The specific torques at 70° and 90° were greater than at 50° (*p* < 0.001 and *p* ≤ 0.007, respectively), and the specific torque at 70° was greater than at 90° (*p* = 0.002). There were no significant changes in muscle cross-sectional area, or MVC at any angle, after endurance training in either age group (Fig. 2a, c).

The lower OD in all muscles of the younger than the older participants (Fig. 2e) (*p* ≤ 0.03) suggests that the intramuscular fat content is higher in the older than in younger participants. Figure 2f shows that the relationship between quadriceps muscle ACSA and VO_2_max is similar pre (*R*^2^ = 0.465 *p* = 0.007) and post-endurance training (*R*^2^ = 0.651, *p* < 0.001), respectively).

### Fibre type composition

**Fig. 3:**
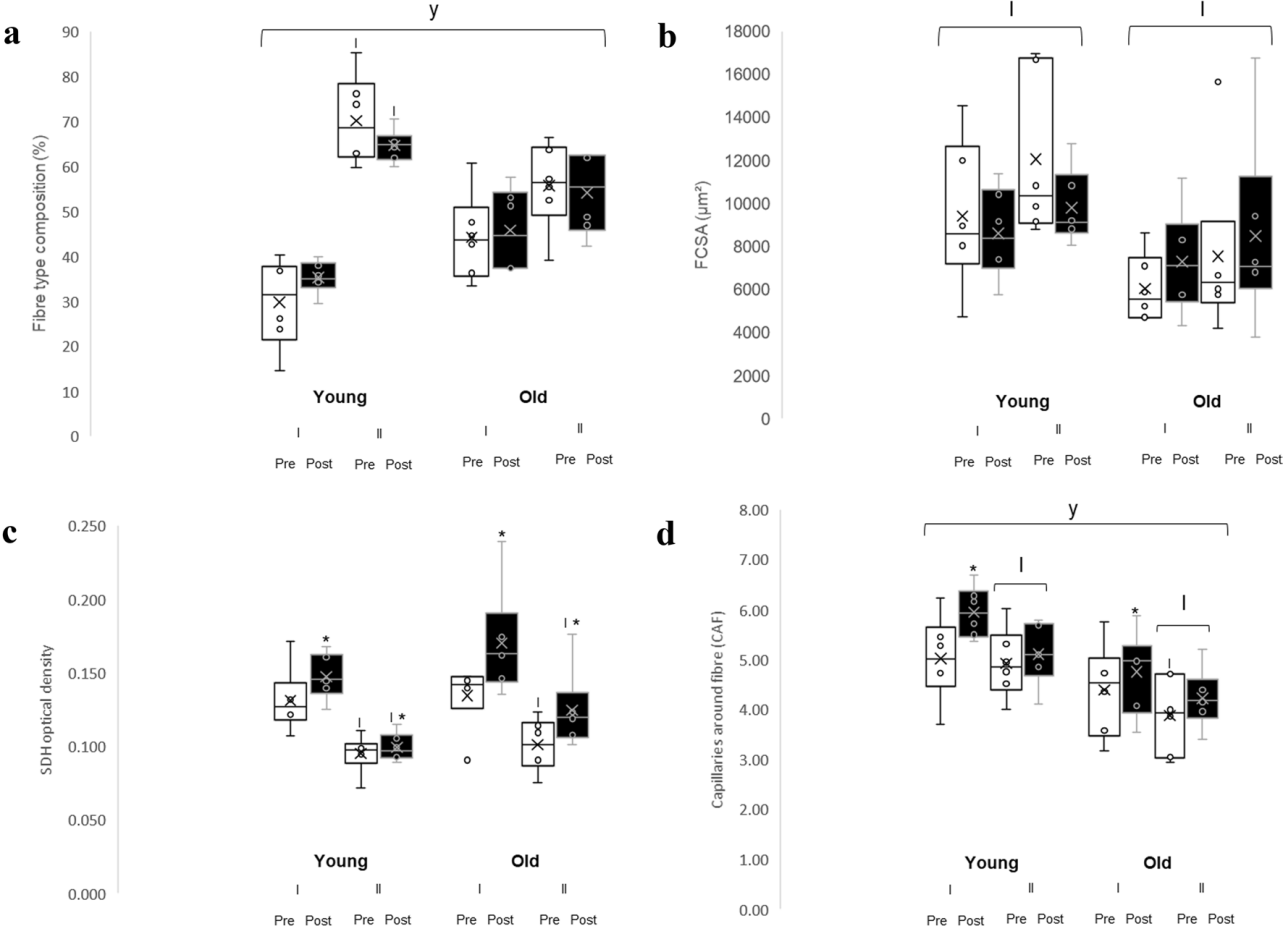
**a** Shows fibre type composition in the *m. vastus lateralis* from young and old highly resistance-trained men pre- and post-endurance exercise. **b** Shows fibre cross-sectional area (FCSA) for type I and type II fibres pre- and post-endurance exercise in young and old highly resistance-trained men. **c** Shows succinate dehydrogenase (SDH) staining optical density for type I and type II fibres pre- and post-endurance exercise in young and old participants. **d** Shows the capillaries around fibres (CAF) for type I and II fibres pre- and post-endurance exercise in young and old participants. *Indicates a training effect at *p* ≤ 0.043; ^y^indicates a significant difference compared to young subjects at *p* ≤ 0.028; ^I^indicates a significant difference compared to type I fibres at *p* ≤ 0.041

Older participants had a larger proportion of type I fibres and a smaller proportion of type II fibres when compared to younger participants (*p* = 0.014) (Fig. 3a). Younger participants had a greater proportion of type II fibres compared to type I (*p* = 0.004) whereas older participants had similar proportions of type I and II fibres. There was no significant effect of superimposed endurance training on fibre type composition.

### Fibre cross-sectional area

There was a main effect of fibre type (*p* = 0.017) without any significant type × age or type × training interactions, indicating that type II fibres were larger than type I, irrespective of age and endurance training (Fig. 3b). Neither training nor age had a significant effect on the FCSA.

### Succinate dehydrogenase activity

The SDH activity was significantly higher in type I compared to type II fibres (Fig. 2c; *p* < 0.001). Endurance training led to an increase in SDH activity in both type I and type II fibres (*p* = 0.019 and *p* = 0.043, respectively) in both age groups (Fig. 3c).

### Capillarisation

Both type I and II fibres had lower CAF in older compared to the younger participants (*p* = 0.028). Type I fibres had a higher CAF than type II fibres (*p* = 0.002) in both younger and older participants. There was a training*fibre type interaction (*p* = 0.034). Post hoc analysis revealed that superimposing endurance training onto resistance training increased CAF for type I (*p* = 0.007) but not for type II fibres (Fig. 3d), irrespective of age.

### Fibre form factor

There were no significant changes in muscle fibre roundness after endurance training in either age group, nor was there a difference between fibre types or between younger and older participants (Table 1).

## Discussion

The main finding of the present study is that superimposing endurance training on a regular resistance exercise programme of highly resistance-trained younger and older men induces an increase in muscle oxidative capacity without a decrement in muscle (fibre) size in both age groups that was accompanied by angiogenesis. This suggests that even in older people highly trained for strength, benefits of endurance exercise do not impair force production or lead to reductions in muscle size.

### Age-related changes in muscles of highly resistance-trained men

We observed that even when maintaining high levels of resistance exercise, there is still an age-related reduction in muscle mass, absolute maximal oxygen consumption (L·min^−1^) and muscle strength. This is also reported in elite master weightlifters where the relative rate of age-related decline in muscle power was similar to that of control subjects, but they still had a larger muscle power compared to age-matched control subjects (Pearson et al. 2002). This phenomenon is not limited to power athletes but the exercise performance in all athletic disciplines shows an age-related decline (Ganse et al. 2018) that in endurance athletes was associated with an age-related reduction in VO_2_max at a similar relative rate to untrained subjects (Tanaka and Seals 2008).

Here, we showed that also in highly resistance-trained men the absolute VO_2_max decreased with age, but the VO_2_max per kg body mass was similar in younger and older participants, suggesting that the decline in VO_2_max with ageing is largely due to a loss of muscle mass (Fleg and Lakatta 1988). This is further supported by our observation of a positive relationship between quadriceps ACSA and VO_2_max.

The lower MVC torque in older compared to younger participants seems to be largely due to a loss of muscle mass. The higher OD of muscle in the MRI images of older participants suggests that they have greater levels of intramuscular fat when compared to younger subjects. The accumulation of intramuscular fat during ageing can reach levels of more than 10% (Schwenzer et al. 2009) and may result in a reduced specific tension that will further contribute to the lower muscle strength (McPhee et al. 2018). Yet, we observed that the specific torque (MVC torque per ACSA) was similar in our younger and older participants. The similar specific force (per ACSA) may be explained by the reduction in pennation angle that accompanies the decrease in muscle size during ageing, where the fascicles are more in line of pull of the tendon (Degens et al. 2009). Alternatively, the maintenance of specific tension in our resistance-trained participants suggests that although resistance training cannot prevent the age-related reductions in muscle size, it may help to maintain the ‘muscle quality’ or force per unit area of muscle (Reeves et al. 2004).

The smaller muscle ACSA was probably more related to a reduction in fibre number that has often been reported during ageing (McPhee et al. 2018) than a decrease in fibre size, as we did not find a significant difference in FCSA between younger and older highly resistance-trained men. Such an absence of an age-related reduction in FCSA was also seen in master endurance cyclists (Pollock et al. 2018), but not in master sprinters (Korhonen et al. 2006). Whatever the cause of the discrepancy, these observations suggest that regular exercise may attenuate the age-related fibre atrophy, but not the age-related loss of muscle fibres, corresponding with the observation that motor unit loss is not attenuated in longstanding master athletes (Piasecki et al. 2019).

We found that the biopsies of our 61- to 77-year-old participants exhibited a greater proportion of type I fibres than the younger group, similar to the increased proportion of type I fibres found in muscles from older people (Larsson et al. 1978). This is, however, an equivocal finding, as others have reported no significant age-related change in the fibre type composition of the *m. vastus lateralis* (Andersen 2003; Barnouin et al. 2017).

Similar to what has been found in the recreationally active population (Barnouin et al. 2017), the capillarisation of the muscles from our older highly resistance-trained men was lower than that found in younger participants. Since blood flow is, via shear stress, an important factor for the maintenance of the vascular bed (Hudlicka et al. 1992), the age-related capillary rarefaction may be due to a reduction in sheer stress resulting from impaired vasodilation and blood flow responses in ageing (Proctor and Parker 2006).

### Is there a concurrent training effect?

Endurance exercise has long been associated with atrophy of type I and type II fibres and an increase in muscle oxidative capacity (Kraemer 1995; Baar 2006; Staron et al. 1984), and therefore has been thought to diminish the resistance training-induced hypertrophy via the so-called concurrent training effect (Hickson 1980). It is possible however, that this is due to non-functional overreaching through excessive training frequencies, intensities and volumes, as others have found comparable outcomes in combined training groups to those subjected to resistance training only when moderate frequencies and intensities were used (McCarthy et al. 1995).

The inverse relationship between fibre size and oxidative capacity suggests that there is a trade-off between fibre size and oxidative capacity (van Wessel et al. 2010; van der Laarse et al. 1998), where due to this trade-off, the endurance exercise-induced increase in oxidative capacity may cause muscle fibre atrophy. Yet, we have seen in rodent studies that this constraint on fibre size may be broken. For instance, hyper-muscular myostatin null mice overexpressing oestrogen-related receptor gamma (Errγ) have a similar fibre size as the myostatin null mice, yet with an elevated oxidative capacity (Omairi et al. 2016), and hypertrophy of overloaded mouse plantaris muscles was accompanied by an increase in oxidative capacity (Ballak et al. 2016). However, the hypertrophied fibres in the muscles of these mice are still smaller (1500 µm^2^ in Ballak et al. (2016)) or the same size (up to 4000 µm^2^ in type IIb fibres; (Omairi et al. 2016)) than untrained human muscle fibres (4000 µm^2^; (Barnouin et al. 2017; Wust et al. 2009)). It could thus be that the size constraint is not yet reached, and that only in highly resistance-trained men with much larger fibres (8000 µm^2^ in our younger group) any increase in oxidative capacity will constrain fibre size and induce atrophy. While we found that endurance training added to the regular resistance exercise of highly resistance-trained men induced an increase in oxidative capacity, this was not accompanied by a reduction in FCSA. These observations challenge the concept of a trade-off between fibre size and oxidative capacity.

There are several potential explanations for this apparent violation of the size constraint, such as a flattening of the fibres to reduce diffusion distances from the periphery to the core of the fibre, increased myoglobin levels to maintain oxygen availability to mitochondria even at low oxygen tension, movement of mitochondria to the periphery of the fibre and/or angiogenesis (Hendrickse and Degens 2019). As in oxidative, more than in glycolytic, fibres mitochondria are more concentrated in the sub-sarcolemmal region (Wust et al. 2009), such a redistribution could also occur when endurance training is superimposed on resistance exercise. However, redistributing mitochondria to the sub-sarcolemmal region creates longer diffusion distances for ATP from the mitochondria to the ATP-consuming myofibrils in the core of the fibre (Kinsey et al. 2007) that could put in turn put a diffusion limit on fibre size (Degens 2012). It remains to be seen, however, whether such a redistribution occurred in our population. We did not see, however, any change in the form factor of the fibres, indicating no significant change in the shape of the fibres, e.g. to a flattened shape to decrease diffusion distances, but we did see a significant increase in the number of capillaries around a fibre, in particular around type I fibres. A similar situation was seen in hypertrophied mouse plantaris where the increase in oxidative capacity and fibre size was accompanied by angiogenesis, and the attenuated hypertrophy in older mice was associated with impaired angiogenesis (Ballak et al. 2016; Hendrickse et al. 2020). In the current study, we did not find evidence for an attenuated angiogenic response in the older highly resistance-trained men, similar to that seen in older women (Gavin et al. 2015). Thus, angiogenesis in our population may well have served to ensure an adequate oxygenation in the face of an increased oxidative capacity and helped to overcome the size constraint in both younger and older highly resistance-trained men.

Muscle capillarisation may well be a determining factor in hypertrophy, as indicated by the attenuated hypertrophy in overloaded muscles from older mice that was associated with impaired angiogenesis (Ballak et al. 2016; Hendrickse et al. 2020) and the lower hypertrophic response to resistance training in muscles with lower capillary density, particularly in older adults (Snijders et al. 2017; Moro et al. 2019). Based on these and the observations in the present study, we propose that endurance training prior to a dedicated resistance-training-only programme may augment increases in muscle mass by preventing diffusion limitations (Hendrickse and Degens 2019).

At first glance, the increase in muscle oxidative capacity without a concomitant rise in VO_2_max post training is difficult to understand, but VO_2_max is limited by the cardiovascular system and not by the working muscle (McPhee et al. 2009). In addition, our data are in line with another study where the change in VO_2_max did not correlate with the change in muscle SDH concentration after an endurance-training programme (McPhee et al. 2011). Here, we thus have muscular adaptations to the superimposed endurance-training programme, but apparently no, or only minimal cardiac adaptations. We have no explanation for this observation, but it could be that the weekly duration of the endurance training programme did not reach the threshold required for cardiovascular and cardiac adaptations to occur (Fagard 2003). Another possibility to induce such adaptations is the use of high-intensity endurance training, but then the risk of CTE may indeed develop (Sousa et al. 2019). Our study shows that endurance training of moderate duration elicits beneficial effects without inducing loss of muscle mass in resistance-trained people.

### Limitations

One limitation is the home-based character of the endurance training, where therefore control of these sessions was minimal. However, we did ask for a report of each session and analysis of these reports showed that each person completed at least 90% of the sessions. Further details on the reasons for missed sessions and whether consecutive sessions were missed would be of benefit to determine the actual exercise dose for each individual (Fairman et al. 2020). While the resistance training protocols were not controlled, all participants completed 4 resistance training sessions per week and had regularly participated in resistance training for a minimum of 5 years.

### Perspectives

Concurrent training in younger and older resistance-trained men led to improvements in oxidative capacity without muscle fibre atrophy, thus providing evidence that the inverse relationship between fibre size and oxidative capacity can be overcome. Additionally, there were no reductions in MVC in both younger and older subjects. Our observations add to evidence that challenges previous assumptions about the “jack of all trades, master of none” nature of concurrent training and support the concomitant use of resistance and endurance training in older people. Indeed, incorporation of endurance training has been found to augment skeletal muscle hypertrophy under certain conditions (Murach and Bagley 2016; Lundberg et al. 2013). As such, carefully considered incorporation of endurance training may provide endurance benefits to both older and younger resistance-trained men without a reduction in muscle size or strength.

## Data Availability

The data that support the findings of this study are available from the corresponding author upon reasonable request.
